# Cellular microenvironment controls the nuclear architecture of breast epithelia through β1-integrin

**DOI:** 10.1080/15384101.2015.1121354

**Published:** 2016-01-28

**Authors:** Apolinar Maya-Mendoza, Jiri Bartek, Dean A. Jackson, Charles H. Streuli

**Affiliations:** a Faculty of Life Sciences and Wellcome Trust Center for Cell-Matrix Research, University of Manchester, Manchester, United Kingdom; b Department of Genome Integrity, Danish Cancer Society Research Center, Copenhagen, Denmark; c Science for Life Laboratory, Division of Translational Medicine and Chemical Biology, Department of Medical Biochemistry and Biophysics, Karolinska Institute, Stockholm, Sweden

**Keywords:** breast cancer, breast mammary gland, cell cycle, cell senescence, extracellular matrix, integrin, nuclear structure

## Abstract

Defects in nuclear architecture occur in a variety of diseases, however the fundamental mechanisms that control the internal structure of nuclei are poorly defined. Here we reveal that the cellular microenvironment has a profound influence on the global internal organization of nuclei in breast epithelia. A 3D microenvironment induces a prolonged but reversible form of cell cycle arrest that features many of the classical markers of cell senescence. This unique form of arrest is dependent on signaling from the external microenvironment through β1-integrins. It is concomitant with alterations in nuclear architecture that characterize the withdrawal from cell proliferation. Unexpectedly, following prolonged cell cycle arrest in 3D, the senescence-like state and associated reprogramming of nuclear architecture are freely reversible on altering the dimensionality of the cellular microenvironment. Breast epithelia can therefore maintain a proliferative plasticity that correlates with nuclear remodelling. However, the changes in nuclear architecture are cell lineage-specific and do not occur in fibroblasts, and moreover they are overcome in breast cancer cells.

## Introduction

Interphase nuclei are sophisticated organelles that contain a number of compartments involved with determining transcript profiles and cell fates. Within the interphase cell, higher order nuclear organization has widespread effects on tissue-specific gene expression, and structural remodeling of the nucleus has a key influence on cell phenotype.^1^ Several nuclear compartments including nucleoli, nuclear speckles and transcription centers have been characterized, and chromosomes are partitioned into discrete territories.^2-6^ However, little is known about the mechanisms that determine the number of nuclear compartments, or how their sub-nuclear distributions and dynamic properties are controlled.^7-9^ In addition, the extent to which spatial nuclear organization defines cell fate decisions is not well established.^10^ Understanding how the internal structure of nuclei is regulated is important because defects in nuclear organization contribute to diseases such as cancer.^11^

Cells in vivo function in 3-dimensional tissues. However, the experimental analysis of mechanisms controlling intracellular processes, including nuclear organization, usually involves planar 2-dimensional cultures of cells on plastic dishes. Contemporary opinion now indicates that the 3D microenvironment within tissues has a profound influence on cell phenotype, by controlling gene expression.^12,13^ This cellular niche includes the extracellular matrix (ECM), soluble factors and other cells, and all of these, together with the dimensionality of the niche itself, determine the fate and phenotype of cells.^14-18^

We therefore hypothesized that one mechanism to explain the link between the microenvironment of a cell and its fate is via a control on the number and function of nuclear compartments.^19^ Here we address this hypothesis using breast epithelia, a paradigm for understanding the molecular basis of cellular differentiation and cancer progression. Using this cell model, we demonstrate that the cellular microenvironment controls the internal architecture of nuclei, and that the mechanism is via a novel form of cell cycle arrest. Moreover, while the link between matrix dimensionality, cell cycle arrest and nuclear architecture operates in normal epithelia, it is uncoupled in breast cancer.

## Results

### Cellular microenvironment dictates the nuclear complexity of breast epithelia

**Figure 1.**
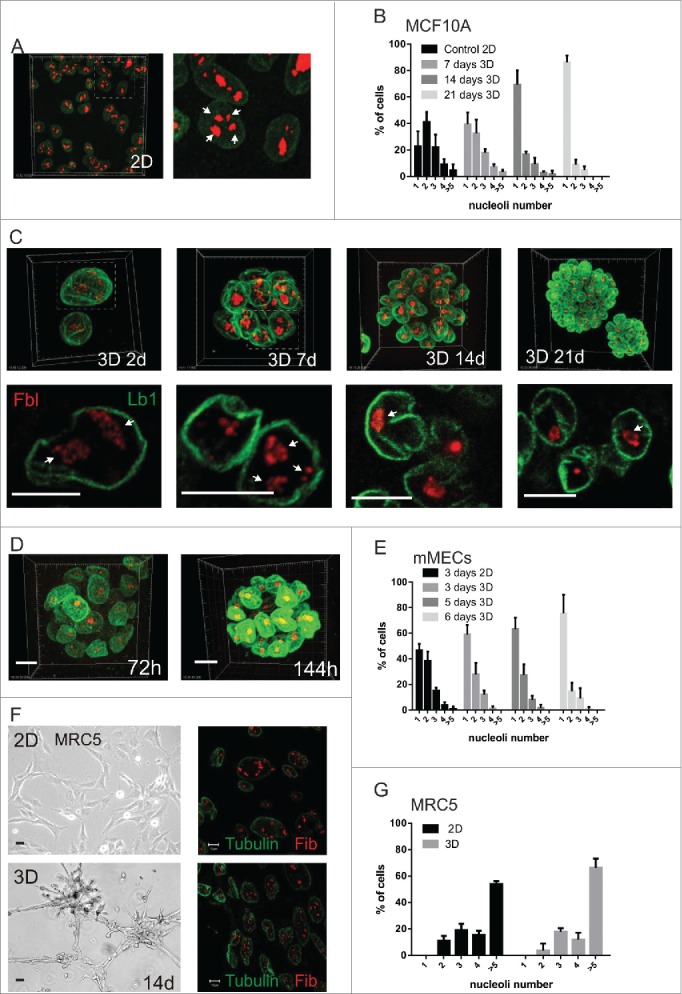
Cellular microenvironment dictates the nucleolar complexity of breast epithelia (A-C) MCF10A. Representative low and high power views of cells in 2D (A) and 3D (C) stained with lamin-B1 (green) and fibrillarin (red); upper images are maximum imaging projections and lower images are high magnification views of confocal slices. The areas enlarged are shown by dotted lines and nucleoli indicated by arrows. The percentage of cells containing 1, 2, 3, 4, or >5 nucleoli in planar culture (2D n = 192 nuclei for this representative experiment from at least triplicates); or after 7, 14 and 21-d on 3D culture on LrECM (n = 172, 177, 205 respectively) are shown (B). (D, E) Primary murine MECs were grown in 3D cultures (D) and the percentage of nuclei with 1, 2, 3, 4, or >5 nucleoli determined (E); nucleoli in cells grown in 2D culture were used as a control (2D: 72-h n = 129; 3D: 72-h n = 184, 120-h n = 141, 144-h n = 129). (F, G) MRC5 diploid fibroblasts were cultured in 2D (upper panels) and 3D (lower panels) for 14-d and imaged using phase contrast (left) and confocal (right) microscopy; confocal images show fibrillarin (red) and tubulin (green) staining. While phase contrast images show that cells undergo cellular rearrangements in 3D culture (F), no difference in nucleolar number was seen when cells were grown in 2D and 3D (2D cultures n = 90, 3D cultures n = 188). Scale bars, 50 μm (phase) and10 μm (fluorescence).

To determine mechanisms controlling nuclear architecture, we compared the distribution and number of nuclear sub-compartments of breast epithelia cultured on planar 2-dimensional substrata (2D culture) and 3-dimensional laminin-rich ECM gels (LrECM) (3D culture). In 2D culture, human MCF10A breast epithelia proliferated to form sheets of cells, which contained multiple fibrillarin-containing nucleoli (Fig. 1A-B). The number of these sub-nuclear compartments was independent of either cell confluence or the type of ECM substrata used (Fig. S1). In contrast to planar culture, cells in 3D culture formed multicellular acini resembling in vivo alveoli (Fig. 1C).^14^ Under these conditions the spatial organization of nuclear compartments became simplified, with the number of nucleoli reducing to one in most cells, by 14–21 days in 3D culture (Fig. 1B-C). Primary mammary epithelial cells isolated directly from mice (MECs), also contained fewer nucleoli in 3D culture than on planar substrata, particularly after 6 days in culture (Fig. 1D-E). These results suggest that the cellular microenvironment determines the internal spatial arrangement of nuclei.

To evaluate whether the dimensionality of the ECM itself determines nuclear complexity, MCF10A cells were cultured on fixed, then washed, LrECM to present cells with a high concentration of laminin in a 2D configuration. In contrast to culture in a 3D matrix, these cells were unable to simplify their nuclei (Fig. S2A-C). Thus matrix dimensionality, rather than the type of matrix protein, provides the local cues for nuclear reorganization.

Nucleoli provide readily quantifiable metrics of global nuclear structure, and although other nuclear compartments such as speckles (Sc-35 labeling) are less easy to monitor, they also changed in response to 3D culture (Fig. S2D,E). Thus the cellular microenvironment has a global influence on nuclear organization. Altered nuclear architecture was specific to sub-nuclear structures because nuclear volume was no different between cells in planar 2D culture and mature 3D acini (Fig. S2F, G).

The tissue-like environment of 3D culture profoundly effects epithelial cell fate. To examine whether the connection between ECM and nuclear organization depends on differentiation, we quantified the number of nuclear sub-compartments in primary MECs cultured in the presence of prolactin, which induces expression of the tissue-specific protein β-casein. There was no difference in the number of nucleoli, revealing that nuclear simplification does not occur downstream of cellular differentiation (Fig. S2H, I). To test if there was a link between transcriptional competence and nuclear compartmentalization, we measured transcription directly by BrU uptake *in situ*. In both MCF10A cells and primary MECs, the amount of transcription was similar in 2D and 3D culture, despite the different nucleolar configuration (http://dx.doi.org/). Moreover, inhibiting transcription in 3D acini did not change the nucleoli number (Fig. S3D, E). This indicates that this aspect of nuclear architecture is not determined by the ability of cells to transcribe RNA.

We also asked if the control of nuclear phenotype by the cellular microenvironment is dependent on cell type. Fibroblasts, which are derived from a different embryonic lineage to breast epithelia, retained multiple nucleoli regardless of whether they were cultured in 2D or 3D conditions (Fig. 1F-G). Thus the local cues to regulate nuclear architecture are cell type-specific.

These experiments demonstrate that cellular microenvironment dictates the nucleolar complexity of breast epithelia, and that this occurs independently of differentiation and without affecting global transcriptional competence.

### Nuclear remodelling in 3D culture occurs downstream of prolonged cell cycle inhibition and induction of a senescence-like state

**Figure 2.**
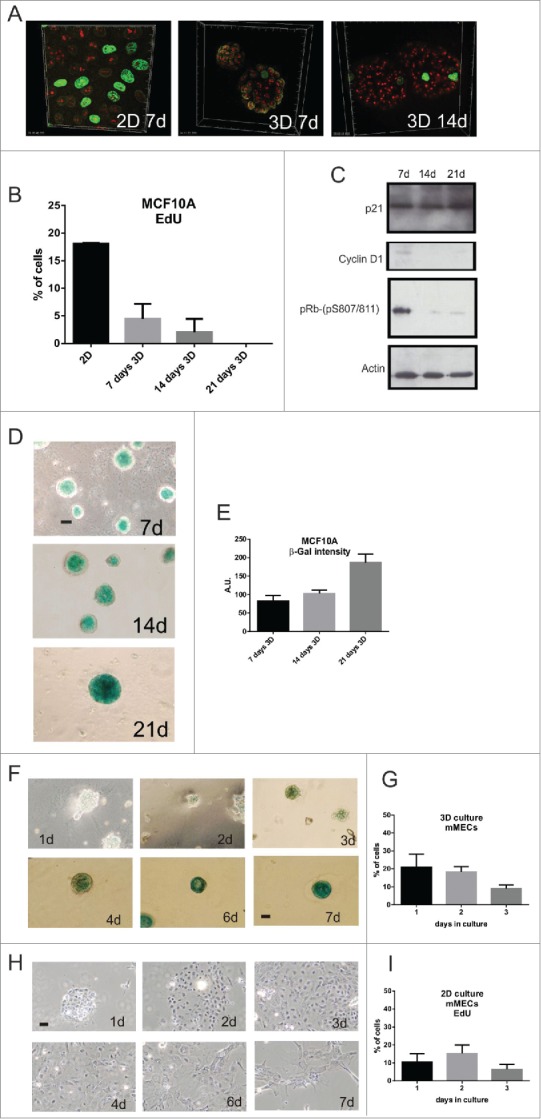
3D culture induces prolonged cell cycle arrest and expression of senescence markers in breast epithelia (A-E) MCF10A proliferation in 2D and 3D culture was assessed by EdU incorporation (green) and nucleoli labeled with anti-fibrillarin (red). Quantification of EdU-positive cells (2D: n = 210 (at 7d of culture); 3D: 7-d n = 186, 14-d n = 179, 21-d n = 240) showed a gradual decline in proliferation in 3D culture (B; S phase index was 23% at 1-d and 5–7% by 7-d). As cells exited cell cycle, expression of the cell cycle regulators cyclin D1 and p-RB declined (immunoblots shown in C) and the senescence markers p21 (C) and SA-β-gal increased (D, E); (D) shows typical phase contrast images on acini stained for SA-β-gal expression and (E) quantification of the stain (n = 25 acini). (F-I) Primary murine MECs grown in 3D (F) and 2D (H) cultures were stained for SA-β-gal activity. Note that no SA-β-gal was detected in 2D cultures whereas expression was clearly seen after 7 days in 3D culture. Changes in cell proliferation in these cultures was measured by EdU incorporation (30 min) after the indicated number of days in 3D (G) and 2D (I) culture. Scale bars, 50 μm.

Since the nuclear remodelling observed in 3D cultures was not transcription-dependent, we reasoned that the mechanism altering nuclear architecture might result from altered cell cycle. The time course of nucleolar simplification in 3D culture (Fig. 1) coincided with a decline of DNA synthesis (Fig. 2A-B) and correlated with the expected changes in cell cycle regulators, including increased p21 expression together with a decrease in cyclin D1 levels and reduced phospho-Rb (Fig. 2C). The cells that ceased to proliferate in 3D culture contained the same number of centromeres as those in 2D, i.e. ∼46 (Fig. S3F-H, p value associated to t test <0.97), showing that they retained their genomic integrity and did not arrest in G2 or M, but rather they accumulated in the G0/G1 phase of the cell cycle.

Prolonged cell cycle arrest in culture is most commonly attributed to a quiescent state that cells assume in G0. However cells cultured as 3D acini could not be stimulated to re-enter cell cycle by the addition of mitogens. Instead, cells expressed senescence-associated-β-galactosidase (SA-β-gal) activity, with levels that increased as the acini matured (Fig. 2D-G). The expression of SA-β-gal, together with a G0/G1 DNA content, Rb activation and inability to be stimulated by mitogens, are hallmarks of cellular senescence.^19^

So in 3D culture, nuclear remodelling in both MCF10A and primary MECs is concurrent with prolonged G0/G1 arrest and the onset of senescence marker expression. In contrast, on 2D substrata primary breast epithelia also exited cell cycle but they did not express SA-β-gal (Fig. 2H-I).^20^ Thus the altered nuclear architecture that results within a 3D tissue-like microenvironment is linked to prolonged G0 arrest in which senescence markers are expressed.

**Figure 3.**
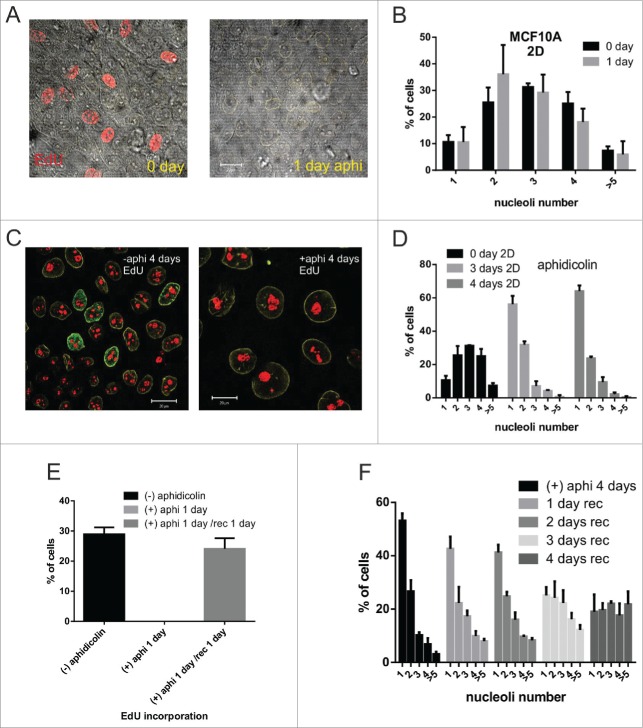
Cell cycle arrest in 2D culture by aphidicolin treatment (A-F) MCF10A cells treated for 24 h with the DNA polymerase inhibitor aphidicolin (1 μg/ml) for 24 h showed no DNA synthesis as judged by EdU (red) incorporation in 2D cultures (A; phase images together with the red EdU channel are shown). Nucleolar number (B) was unchanged over this time frame (CT n = 239, aphidicolin treated cells n =284), but simplified during longer treatments (C,D; n > 250 cells per time point). 2D-cultures treated with aphidicolin for short periods of time (less than 1   day) recovered proliferative capacity rapidly (cells were pulse labeled for 30min with EdU) once the inhibitor was removed (E; n > 250 cells per sample). Following prolonged (4d) cell cycle arrest, cells grown in fresh medium gradually recovered their normal distribution of nucleoli (F; 4-days with aphidicolin: n = 323; 1-day without aphidicolin: n = 285, 2-days n = 457, 3-days n = 359, and 4-days n = 477). Scale bars, 20 μm; note that after 4 days of aphidicoli-induced cell cycle arrest (C) cells are flattened and enlarged, consistent with a senescent state.

To determine whether cell cycle arrest and senescence could cause altered nuclear architecture, we treated cells in planar 2D culture with aphidicolin. The growth arrest induced by inhibition of DNA synthesis over short (1-day) time frames did not reflect the changes in nuclear compartmentalization seen in 3D culture (Fig. 3A-B). However, prolonged inhibition (up to 4 days) resulted in changes of nuclear organization that almost precisely phenocopied those seen in 3D (Fig. 3C-D). After removing aphidicolin, the cells in 2D culture initiated proliferation ^21^ and reverted their nucleoli number (Fig. 3E-F).

**Figure 4.**
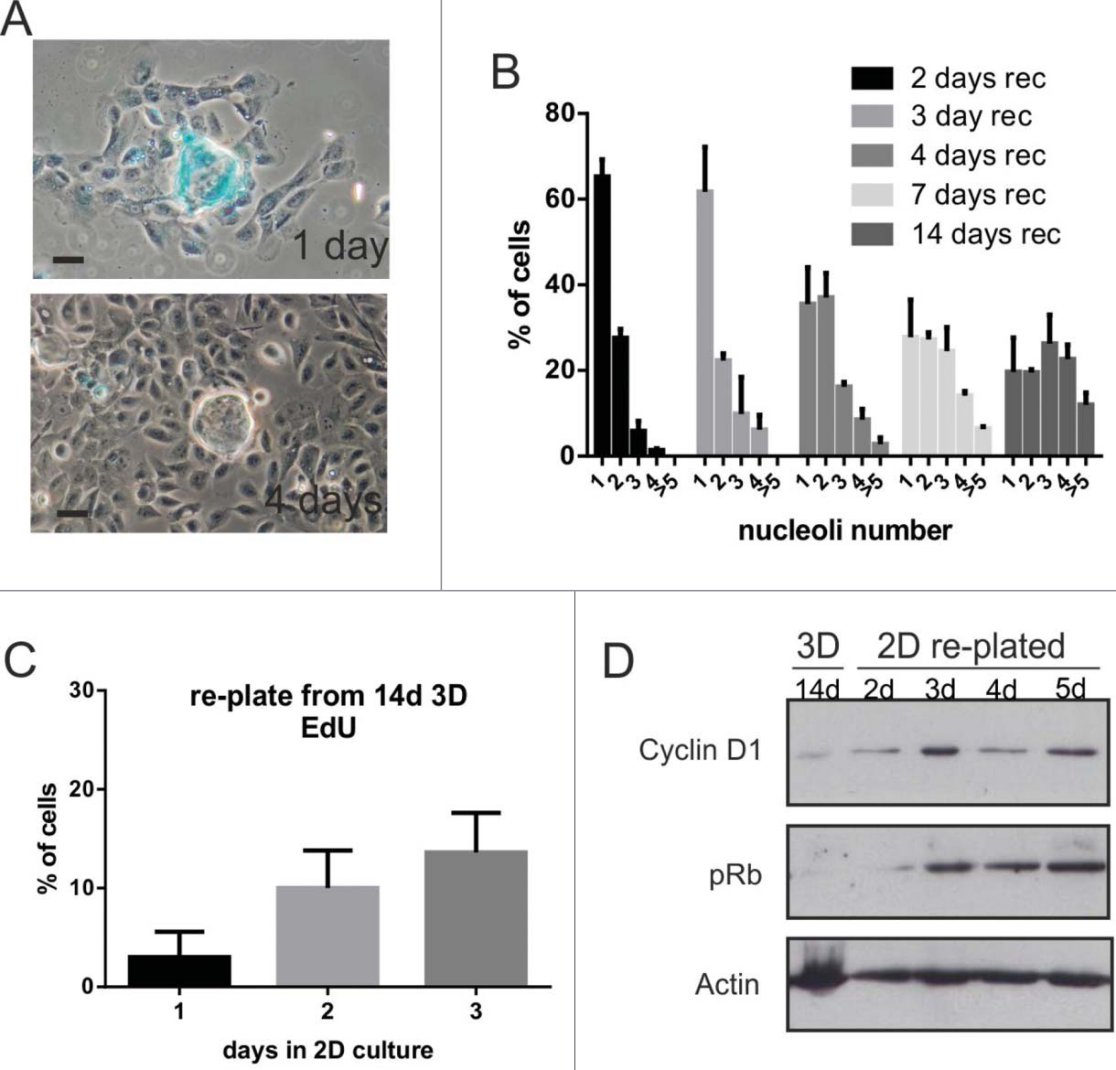
Breast epithelia emerge from arrest in response to changes in their local microenvironment (A-D) MCF10A. To alter the cell microenvironment, acini that had been cultured in 3D for 14-d were picked and re-plated in 2D planar culture. The acini, which initially contained SA-β-gal-staining positive cells, spread onto the plastic (A) and SA-β-gal staining declined as cells emerged onto the 2D substratum. Over a period of 4 d following re-plating, cells showed a progressive increase in nucleolar number (B); n > 250 for each time point), and increased their proliferative capacity, as judged by EdU incorporation (C); 24-h after replate: n = 303; 48-h n = 381; 72-h n = 212). These changes correlated with increased expression of the cell cycle regulators cyclin D1 and pRb (D). Note that primary murine MEC acini that expressed SA-β-gal similarly reverted from cell cycle arrest on changing their microenvironment (Janes et al. 2011). Scale bars, 50 μm.

In view of this plasticity with which breast epithelial cells respond to cell cycle arrest, we determined whether the proliferation of growth-arrested 3D acini could be re-started by altering the cellular microenvironment. When 14 day-old acini were explanted into fresh planar cultures, cells migrated from the acini and lost SA-β-gal expression (Fig. 4A). This transition correlated with the restoration of higher nucleolar numbers (Fig. 4B) and cell cycle re-entry (Fig. 4C-D). Primary MECs, like MCF10A cells, were also able to escape from 3D-induced cell cycle arrest and the apparent onset of senescence when transferred to planar substrata.^20^

These experiments demonstrate that the cellular microenvironment determines the proliferation fates of breast epithelia. The nuclear remodelling induced by 3D culture arises from a prolonged cell cycle restriction that promotes a nuclear state in which cells express the hallmarks of cellular senescence.

### Nuclear remodelling in a 3D microenvironment requires β1-integrins

To explore the mechanism connecting cellular microenvironment with nuclear architecture, we reasoned that this involves ECM receptors, which play a critical role in regulating cell fate decisions.^17^ The formation of mammary acini requires cell-ECM interactions via β1-integrins, raising the possibility that they may also dictate the nuclear organization observed in 3D culture.^22^

**Figure 5.**
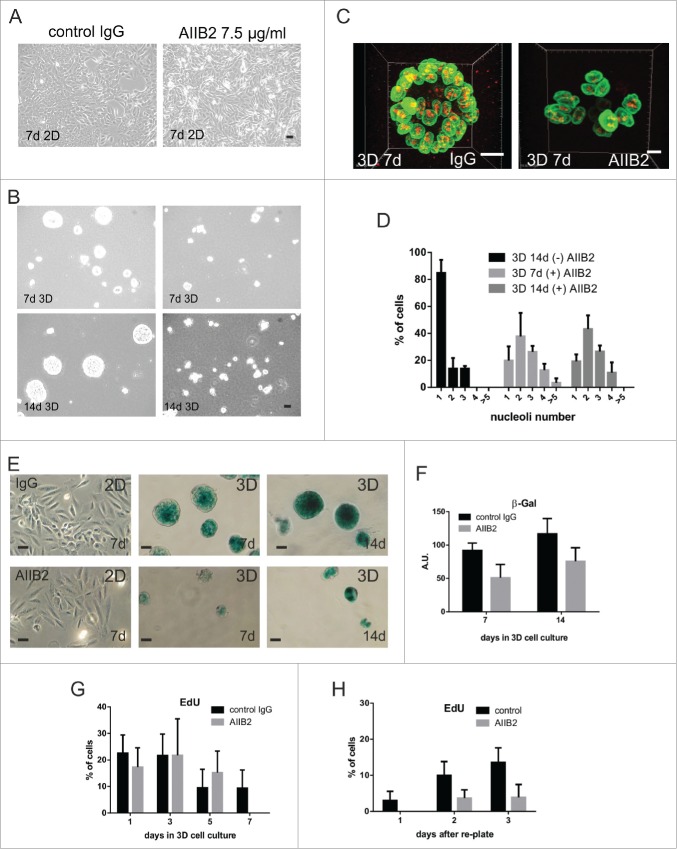
β1-integrin blocking antibody prevents nucleolar simplification (A-D) MCF10A cells were cultured in 2D (A) and 3D (B) in the presence or absence of anti-β1-integrin AIIB2 antibody and examined by phase contrast microscopy over 14 days. During this period, AIIB2 had no effect on cells grown in 2D culture, but it prevented the formation of acini in 3D culture, even though very low levels of apoptosis were seen. The disruption of acinus formation was evident when 3D cultures (C) were stained for laminB1 (green) and fibrillarin (red) and this disruption correlated with the loss of nucleolar simplification that was seen in control mature acini (D; CT; n = 176; AIIB2; 7-d n = 98 and 14-d n = 146). Nucleolar numbers in 3D cultures incubated with isotype IgG mimicked those seen in untreated controls whereas cultures treated with AIIB2 had nucleolar profiles similar to cells grown in 2D culture (see Fig. 1B). The maturation of acini treated with isotype control IgG correlated with increased expression of SA-β-gal (E, F), whereas no expression was seen in 2D cultures and much reduced expression was seen in cultures treated with AIIB2 (E, F), implying that the normal function of β1-integrin was necessary to maintain expression of the senescence marker. The proliferative capacity of cells in 3D culture declined (G) as judged by EdU incorporation, whether or not AIIB2 was present. However, mature acini formed under control conditions returned to cycle much more efficiently than AIIB2-treated acini on re-plating into planar cultures (H; images shown in Fig. S6). Scale bars, 10 μm.

We tested this hypothesis using the function-blocking anti-β1-integrin AIIB2 antibody. AIIB2 did not affect growth of MCF10A in planar culture (Fig. 5A). However, in 3D culture it inhibited acinar formation (Figs. 5B and S4A),^23^ and nuclear remodelling as defined by nucleolar simplification (Fig. 5C-D). Thus β1-integrin function is required directly for nucleolar simplification to occur. Despite the different appearance of cell clusters in the absence and presence of AIIB2, these changes had no significant effect on replication during the early stages of 3D culture (Fig. 5G). In fact, the antibody delayed the onset of SA-β-gal staining in 3D acini (Fig. 5E-F), correlating with the prolonged retention of complex nucleolar patterns. In acinar replating experiments, cell proliferation also required β1-integrins, because the proliferation of 14 day-old acini explanted into fresh planar cultures in presence of blocking antibody AIIB2, was severely affected (Figs. 5H and S4B).

These experiments demonstrate that the characteristic nuclear architecture of breast epithelial cells, which arise in a 3D microenvironment, requires β1-integrins. Moreover, the cells do not need polarity or the formation of multi-cellular acini in order to reorganize their nuclear compartments.

### The control on nuclear organization by cellular microenvironment is uncoupled in breast cancer

**Figure 6.**
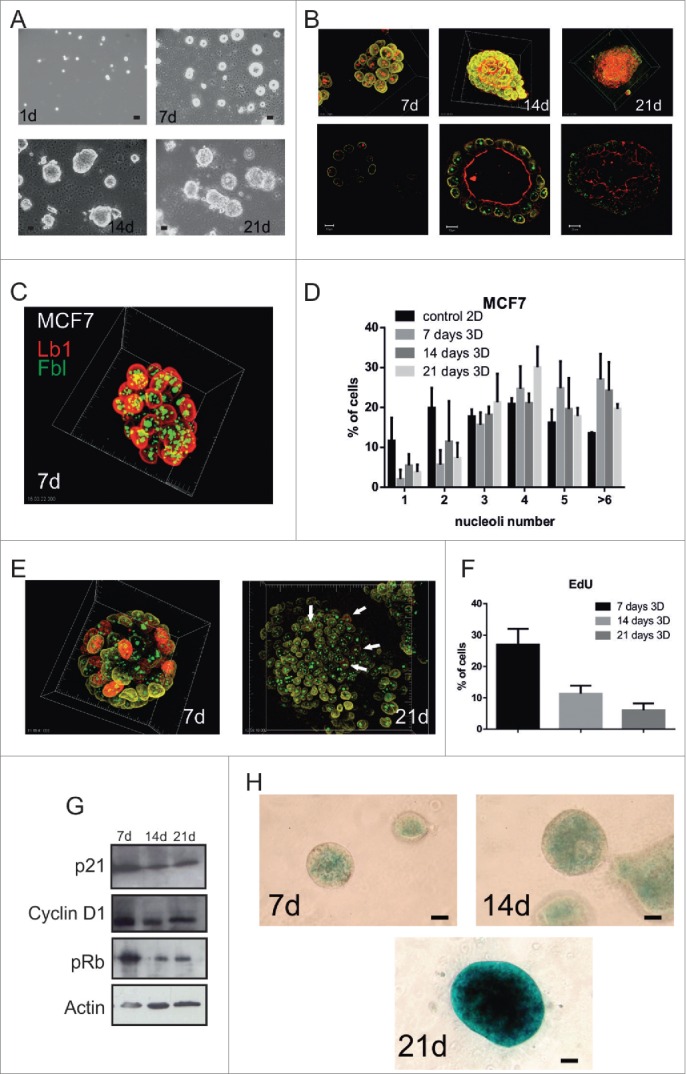
The ability of the cellular microenvironment to control nuclear architecture is uncoupled in breast cancer cells. (A-H) MCF7 cells grown in 3D cultures on LrECM for up to 21-d formed acinus-like structures as judged by phase contrast (A) and confocal microscopy (B). The nuclear envelope (lamin B1, yellow), the fibrillarin centers (red at 7-d, and green at 14 and 21-d) and lumen formation (phalloidin red at 14-d and 21-d only), are shown in projections (upper panels) and confocal sections (lower panels). Note that phalloidin, which stains f-actin, mainly localizes to the apical cell surface and demarcates the boundary of the lumen. Confocal projections (C) of cells cultured in LrECM and stained for nucleoli (green) and lamin B1 (red) were used to monitor changes in the number of nucleoli over 21 days in 3D culture and compared to 2D planar cultures as controls (D; n > 250 for each time point). Throughout this time course, proliferation in 3D culture was assessed by EdU incorporation (E-F; n > 250 for each time point; arrows indicate example cells in S phase). Note that unlike MCF10A cells, transformed MCF7 cells in 3D culture fail to simplify their nucleoli, maintain proliferative capacity and form highly disordered acini. Correspondingly, the expression of cell cycle regulators (cyclin D1 and pRB) and senescent markers (p21 and SA-b-gal) are only partially altered (G, H). Note that there is almost no SA-β-gal staining at 7-d and 14-d, in comparison with the staining in MCF10A (Fig. 3C), and expression is delayed at 21-d. Scale bars, (A and H) 50 μm, (B) 10 μm in left and middle pictures, 20 μm in right picture.

Well-defined 3D cellular organization is implicit for the function of normal tissues, but becomes perturbed in cancer. To determine if the relationship between cellular microenvironment and nuclear architecture is altered in breast cancer, we examined MCF7 cells. As with normal breast epithelia, MCF7 cells proliferated in 3D culture to form multicellular acini (Fig. 6A). Acinar formation was also dependent on β1-integrin signaling (Fig. S4C). In contrast to the non-cancerous cells, MCF7 acini were hollow initially, but eventually formed solid cell masses (Fig. 6B). Moreover during prolonged 3D culture, MCF7 nuclei retained complex nucleolar patterns (Fig. 6C-D). Thus the link between cellular microenvironment and nuclear simplification is broken in these breast cancer cells.

To determine the mechanism behind this uncoupling, we examined cell cycle and senescence markers. Even after 21-d of culture, when MCF10A had exited the cell cycle (Fig. 2A), MCF7 cells continued to proliferate slowly, and they maintained expression of cyclin-D1 and pRb (Fig. 6E-G). Moreover, MCF7 cells did not express SA-β-gal until 21d of culture (Fig. 6H), but even after this extended period their nuclei retained complex nucleolar patterns (Fig. 6D).

To determine how senescence markers are linked to nuclear complexity in breast cancer, we treated planar 2D cultures of MCF7 cells with aphidicolin. Under these conditions, cells expressed SA-β-gal and their nuclear area became larger, indicating a senescence-like phenotype.^21^ However, in contrast to the alteration of nucleolar organization seen when non-transformed MECs cells were blocked in S-phase, MCF7 cells treated with aphidicolin for 4-d were not able to simplify their nucleoli (Fig. S4 D, E).

These experiments demonstrate that in contrast to normal epithelia, the ability of the cellular microenvironment to control nuclear organization is uncoupled in breast cancer cells.

## Discussion

### The cell niche and cell cycle arrest

In this paper we reveal that the cellular microenvironment has a profound influence on the global internal organization of nuclei in breast epithelia. In a 3D microenvironment the cells enter a proliferation arrest that is different to that seen in 2D culture. On planar 2D substrata, primary MECs stop cycling as they reach confluence but do not become senescent and their nuclei do not undergo internal remodelling. In 3D but not 2D culture, mammary epithelia enter a prolonged cell cycle arrest, during which their nuclei reduce nucleolar complexity and alter the organization of other internal compartments such as SC35-rich nuclear speckles.^24^

Integrins have a central role in regulating the metazoan cell cycle, and are involved with G1, metaphase and telophase.^17,25,26^ Here, we show that they also influence nuclear interphase reorganization. Since prolonged cell cycle arrest and nuclear simplification occurs in cells cultured in 3D, but not a 2D configuration in which the matrix has low elasticity, the requirement for integrins may be to detect appropriate extracellular tension. For example, nanoscale forces within the ECM have a profound influence on cell fate by altering cytoskeletal dynamics and tension-dependent signaling pathways.^13^

One possible mechanism is that ECM forces regulate the size and composition of adhesion complexes, and thereby alter nuclear signaling. Stiff substrata, e.g. 2D culture dishes, promote the assembly of large adhesion complexes, which differ in their signaling and cytoskeletal assembly potential from those forming on soft LrECM.^27^ Integrin adhesions harbour several proteins that are also required for cell cycle regulation. For example, integrin-linked kinase and NEDD9 are adhesion complex proteins in interphase and centrosome proteins during mitosis.^28,29^ Thus, altering the ECM niche might directly control cell cycle states in breast epithelia by mobilizing proteins that are otherwise sequestered within adhesion complexes.

Our new work showing the relationship between niche and prolonged cell cycle arrest broadens the niche's role as a central determinant of fate decisions. Proliferating cells can exit the cell cycle either transiently by entering a quiescent state in G0, or permanently as they become senescent.^30,31^ Quiescence can persist almost indefinitely, before cell cycle re-entry is induced by changes in local environment, such as the availability of growth factors. In contrast, senescent cell cycle arrest is considered to be irreversible in normal cells suggesting an end point process during differentiation.^32^

Here we have discovered that breast epithelial cells can assume a ‘resting’ state in which senescence markers are expressed, but from which they can emerge in response to changes in the local microenvironment. In 3D culture, breast epithelia withdraw from the cell cycle, previously shown to correlate with re-organization of the telomere-associated protein, TIN2.^33^ Moreover the cells express SA-β-gal and they develop a senescent phenotype that correlates with changes in nuclear organization.^34^ However, even though cells within 3D culture are unresponsive to changes in growth factors, we find that mammary epithelia are able to re-enter the cell cycle and grow normally, when their microenvironment is changed by transferring cells to planar culture. Interestingly, a long-term inhibition of DNA polymerase, and hence cell cycle progression, resembled the nuclear alterations that were seen in 3D culture. Hence, breast epithelial cells establish a novel form of cell cycle arrest that has some features in common with the senescent phenotype. Since this is not the classic form of irreversible senescence, nor senescence from which variant cells evolve,^35^ it represents perhaps a novel form of reversible G0 arrest.

Although nuclear compartments are different in cells cultured in 2D vs 3D ECMs, there is no change in their competence to transcribe RNA because the rate of BrU incorporation is similar and inhibiting transcription does not change the nucleoli number. Even so, the profiles of gene expression in 2D vs 3D are very different, reflecting different cell contexts and the availability or activity of tissue-specific transcription factors downstream of signaling pathways.^16^ In this regard, global nuclear organization, and specifically the nucleoskeleton, influences chromosomal positioning within the nucleus and contributes to tissue specific patterns of gene expression.^36,37^ Future studies will determine if changes in global chromosome organization are involved with the induction of reversible G0 arrest in breast epithelia.

### Uncoupling the microenvironmental control of nuclear architecture in breast cancer

Our data suggest that one mechanism contributing to tumor progression may be the failure of cells to orchestrate the appropriate phenotypic changes that determine the internal organization of nuclei. Dysmorphology is one of the earliest markers of cancer, being replaced in later stages of the disease by stromal invasion and malignancy. Here we found that the microenvironmental control of nuclear organization is uncoupled in breast cancer cells, suggesting that one mechanism contributing to tumor progression is the failure of cells to induce the appropriate phenotypic changes that determine nuclear internal organization. This has implications for understanding mechanisms of carcinoma progression, because deregulated nuclear architecture contributes not only to aberrant patterns of gene expression in tumors, but also to genomic instability and malignant cancer progression.^11,38^

In summary, we show here that breast epithelial cells have a surprising plasticity in their response to cues from the local environment at the level of nuclear remodelling. Our observations provide new ways of thinking about how the cellular microenvironment influences cell behavior. We also suggest that broken connections between the environment of the cells and their nuclear structure may profoundly influence the course of cancer progression.

## Materials and Methods

### Animals

Mice were housed and maintained according to the University of Manchester and UK Home Office guidelines for animal research. Experiments were conducted according to S1 killing of the Animals Scientific Procedures Act 1986.

### Cell culture

MCF10A cells were grown in DMEM/F12 media with 5% horse serum, L-glutamine, antibiotics, 0.5 μg/ml hydrocortisone, 10 μg/ml insulin, and 20 ng/ml EGF. Primary MECs were isolated and cultured from 18-d pregnant mice and cultured in Ham's F12 medium (LONZA), containing 5 μg/ml insulin, 1 μg/ml hydrocortisone, 3 ng/ml EGF, 10% foetal calf serum, 50 U/ml penicillin/streptomycin, 0.25 μg/ml fungizone and 50 μg/ml gentamycin, and the media was changed every 2 days.^39^ For MCF10A 2D cultures, experiments were done when the cells were <80% confluent. With higher confluence, proportional higher levels of cell apoptosis were seen. For 3D culture, single cells were plated and grown to allow formation of hollow acini, which after 2 weeks remained stable, with no further proliferation despite media changes.^40^ For primary MECs, organoids were plated from the tissue prep. In 2D, the cells proliferated to form confluent monolayers over 4-d and then the cultures ceased proliferating and the cells began to apoptose.^20,39^ In 3D, they proliferated over 4-d and then formed hollow acini that remained stable for at least 1-week.

MRC5 cells were grown in MEM, L-glutamine, sodium pyruvate, non-essential amino acids, 10% FBS and antibiotics. In some experiments, cells were incubated with 1 μg/ml aphidicolin.

MCF7 cells were grown in DMEM media supplemented with 10% FBS, L-glutamine and antibiotics cells plated for 2D cell cultures were grown directly in 35 mm glass base dishes (IKAWI, Japan), for 3D cultures the glass was coated with 50 μl of growth factor reduced LrECM (BM-matrix, BD biosciences) and cell plated at 5 × 10^4^ cells/ml. Next day 3D cultures were overlaid with 2% of BM-matrix in DMEM/F12 media. Media changes were carried out every 4-d.

Blocking antibody AIIB2 against β1-integrin was purified from the commercial hybridoma (Developmental Studies Hybridoma Bank, University of Iowa) using the purification kit Prosep-G (Millipore). The buffers were prepared without sodium azide. Different concentrations of antibody were tested and the final concentration used was 7.5 μg/ml. For control experiments we added purified rat-IgG isotype control.

For differentiation experiments, primary MECs were cultured in 3D for 3-d, then incubated in differentiation media (DMEM/F12 supplemented with insulin and hydrocortisone) containing prolactin (Prl) at 3 μg/ml for 24-h.

2D cultures on thick LrECM were performed as follows; glass base dishes were coated with LrECM as for 3D cultures. The LrECM was fixed using 2% glutaraldehyde in PBS for 1-h at RT. ^41^ The dishes were rinsed 3 x in PBS containing 0.1 M glycine at pH 7.4 and left overnight in this solution. Next day the dishes were washed 3 x using fresh media and left to equilibrate with media for 1-h before plating cells.

For 3D to 2D cell culture re-plate experiments, acini from 14-d in LrECM were recovered using PBS-EDTA.^42^ 15-min incubation and 3 washes in PBS-EDTA were used. Complete dissociation of LrECM was monitored before plating the acini on glass bottom dishes.

### Immunofluorescence

Immunofluorescence was performed on 2D and 3D cultures after 10-min fixation in 4% FA in PBS at RT. For 2D cultures the cells were permeabilized in 1% Triton X100 in PBS. 3D cultures were permeabilized 30 min 1% Triton for intranuclear proteins and 5-min for membrane attached proteins. After permeabilization the samples were washed 3 x in PBS, 3 x in PBS+ (PBS, 0.1% Tween and 1% albumin (SIGMA)), and blocked for 1h in PBS+. The antibodies used were mouse anti-Fibrillarin (Cytoskeleton), rabbit anti-LaminB1 (Abcam), mouse anti-Sc-35 (Sigma), mouse anti-Tubulin (Sigma), mouse anti-CENP-A (Abcam), mouse anti-smooth muscle actin (SMA, Sigma), mouse anti-β-Casein.^43^ The secondary detection was with either Alexa- (Invitrogen) or Cy3-conjugated (Jackson) antibodies. F-actin was detected with phalloidin-TRITC (Sigma). Cells on the glass base dishes were covered with 100 μl of Vectashield (Vector labs).

### Transcription assay

Nascent transcripts were visualized following incorporation of BrUTP in vitro.^44^ Cells on coverslips were permeabilized using Triton X-100 (0.1% for 1-min at 20°C) and transcription performed in buffer supplemented with 500 μM BrUTP, GTP, CTP and 2 mM ATP (5-min at 37°C). BrUTP detection in nascent RNA was performed using a sheep anti-BrdU antibody (Biodesign; M20105S; 1/1000; 1-h; 20°C) followed by Cy3- or Alexa-Fluor-488-conjugated donkey anti-sheep IgG (1/1000; 30-min; 20°C). The intensity of staining was measured using Image J software. To inhibit transcription, cells plated were incubated with 0.2 μg/ml Actinomycin D (Sigma) and added to the culture media for 1.5 or 2-h.

### Immunoblotting

Proteins were extracted from cells using 2x SDS loading buffer. For 3D cultures the BM-Matrix was dissolved in PBS-EDTA ^45^ and released cells suspended in 2x SDS loading buffer. Equal amounts of protein were used (12–15 μg of total protein). Equivalent loading was assessed by referral to β-actin. The primary antibodies used were, p21 (2946, Cell Signaling), Cyclin D1 (2926, Cell Signaling), pRB (Ser807/811, Cell Signaling), and β-actin (A1978, Sigma).

### Cell proliferation and senescence

Cell proliferation was measured by incorporation of either 25 μM BrdU or 10 μM EdU for 30-min. For BrdU detection, cells in 2D and 3D cultures were fixed as for immunofluorescence, and treated with 2.5 M HCl.^46^ EdU detection was carried out according to the supplier's method (Invitrogen).

SA-β-Gal staining was performed using the BioVision Senescence detection kit following the manufacturer's protocol. Signal quantification was determined using Image J software.

### Microscopy and image analysis

Different microscopes were used in this study. Some samples were imaged using a LSM 510, which was the best microscope for low ratio noise/signaling. For long set of measurements in 2D cultures (nucleoli number, nuclear size, etc), we used an Axioplan-200M (Zeiss) microscope with automated stage and focus. For high speed we used a fully automated DeltaVision system. Color microscopy was performed with an Axiovert 40 (Zeiss) with a 10 Mpixel digital camera (Nikon). Area and volume size were quantified using LSM software analyzer and ImarisTM software. 3D reconstruction from Z-stack were carried out using ImarisTM software, Gaussian filtering was applied using the value suggested by the software.

## Supplementary Material

2015CC6907-f02-z-4c.pdf
