# Supplementary figures and images for: Cellular microenvironment controls the nuclear architecture of breast epithelia through β1-integrin
Source: Cell Cycle. 2016 Jan 28;15(3):345–56. doi: 10.1080/15384101.2015.1121354 (PMC4943696; doi:10.1080/15384101.2015.1121354)

A

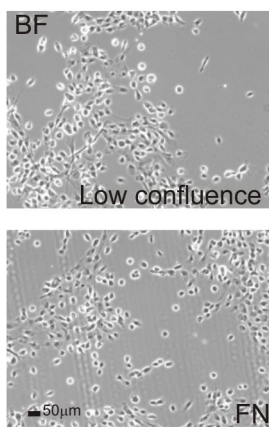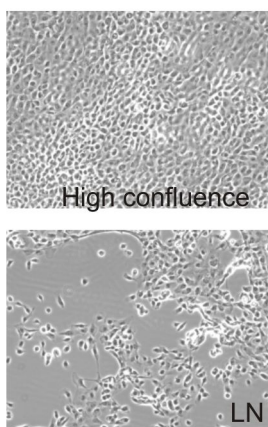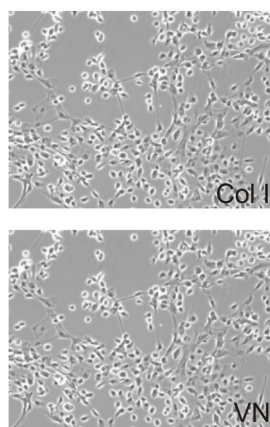

B

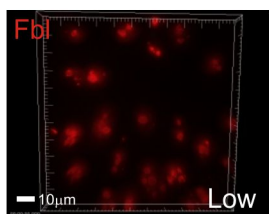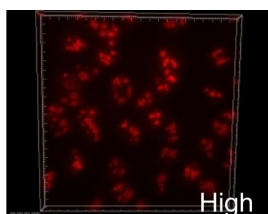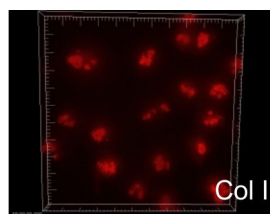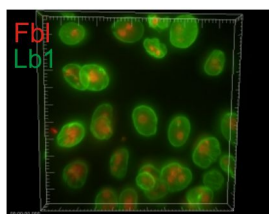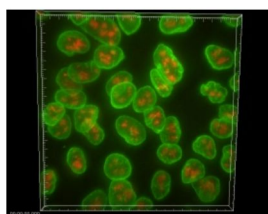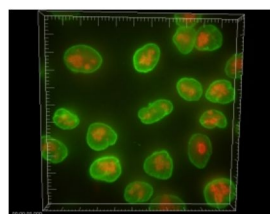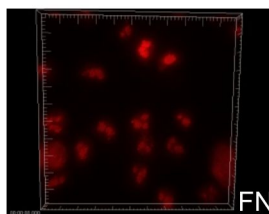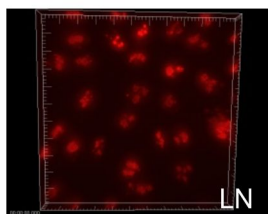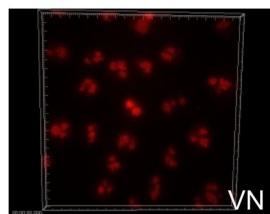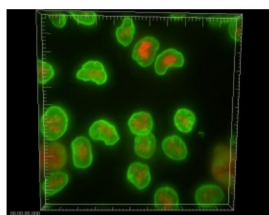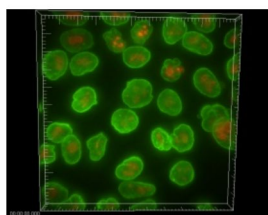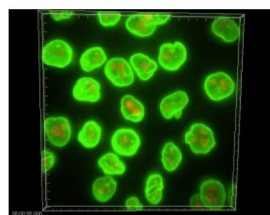

C

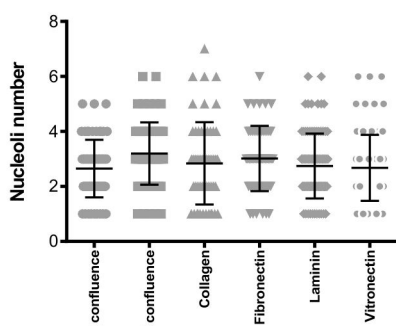

D

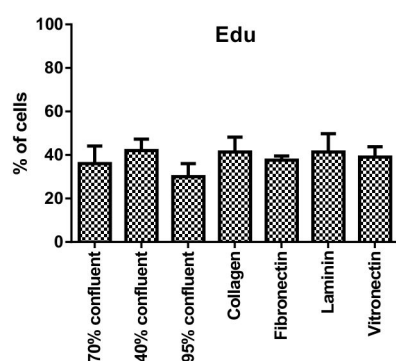

A

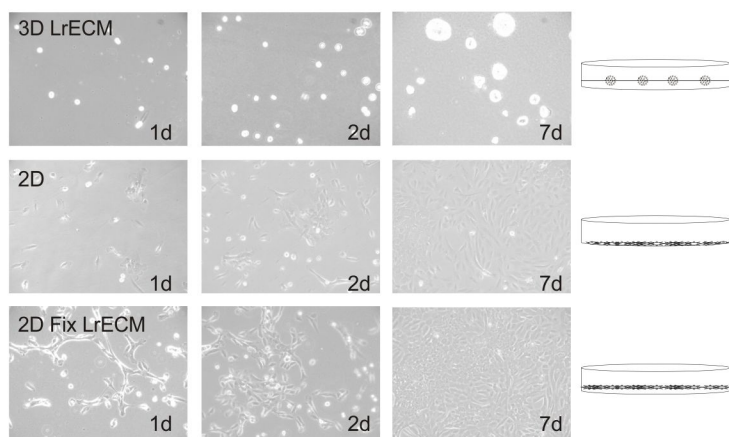

B

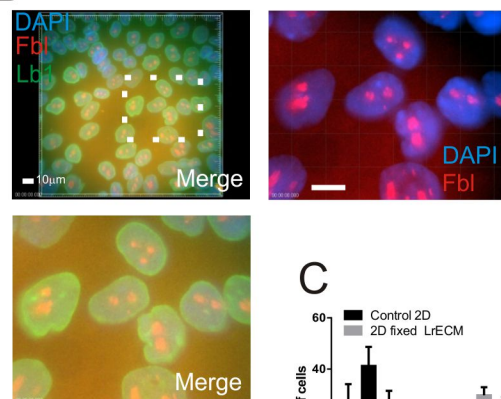

C

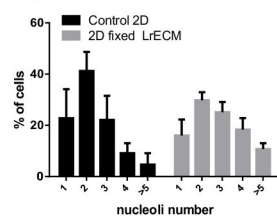

D

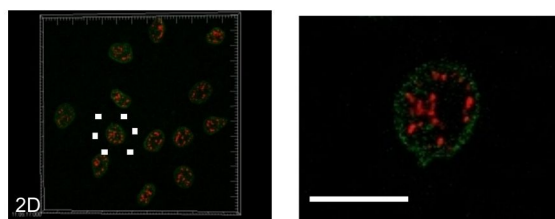

E

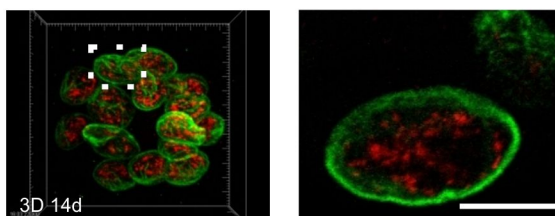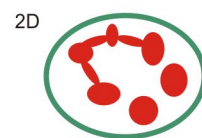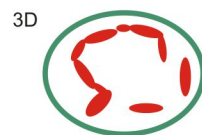

F

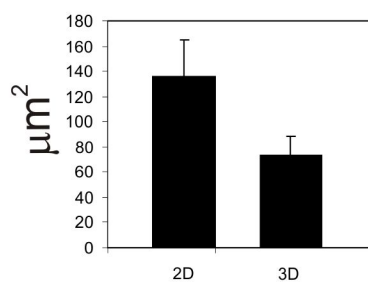

G

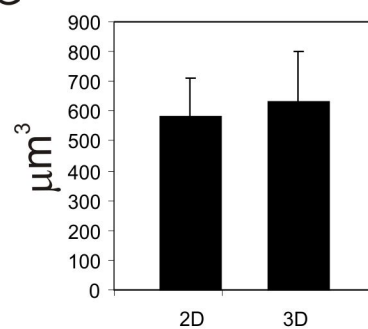

H

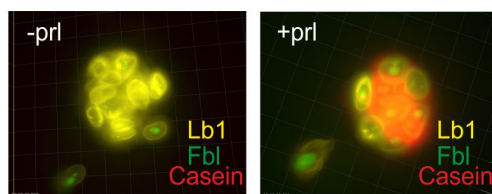

I

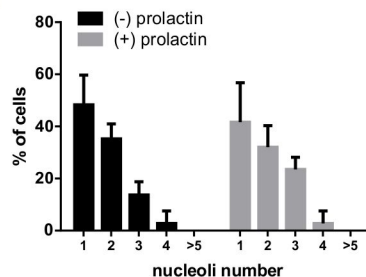

Supplemental figure S2.

A

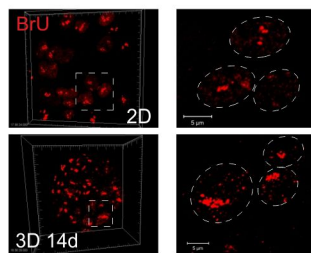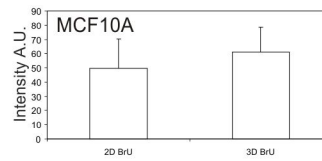

B

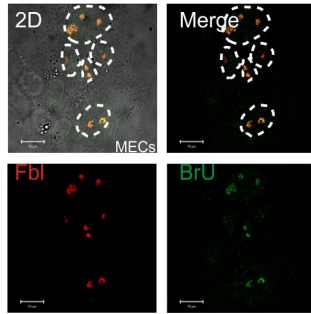

C

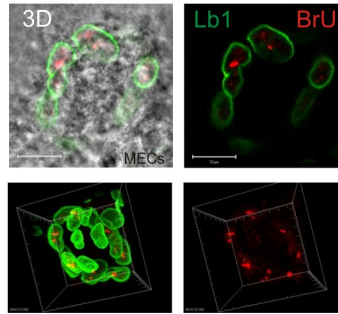

D

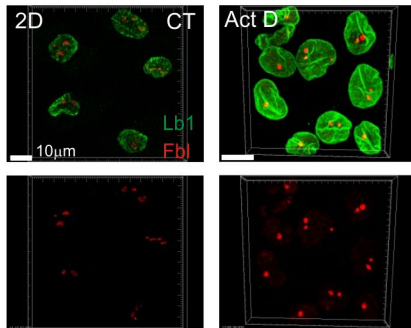

E

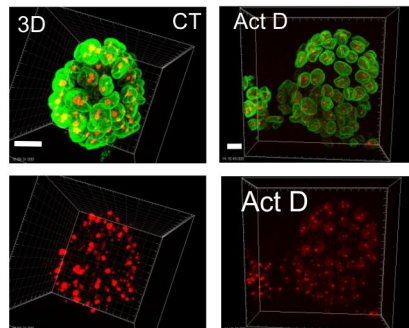

F

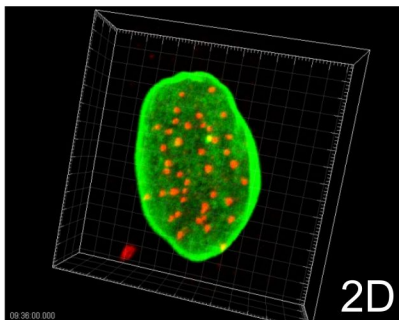

G

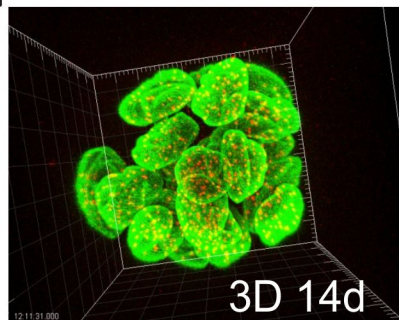

H

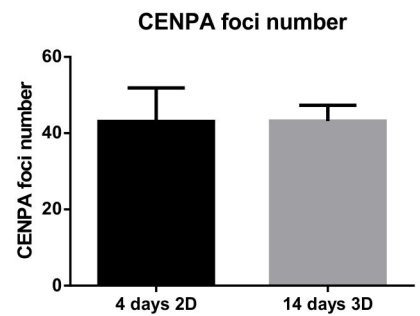

Supplemental figure S3.

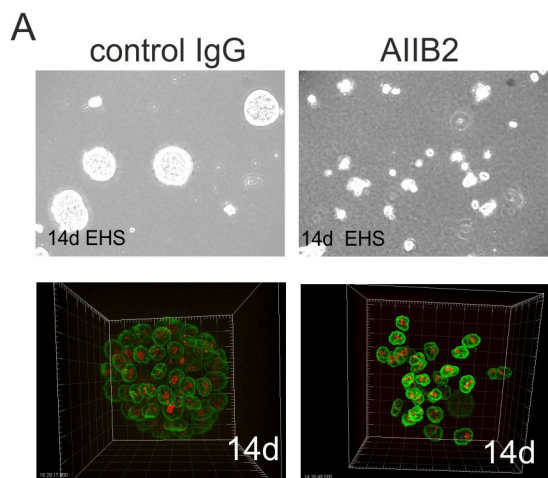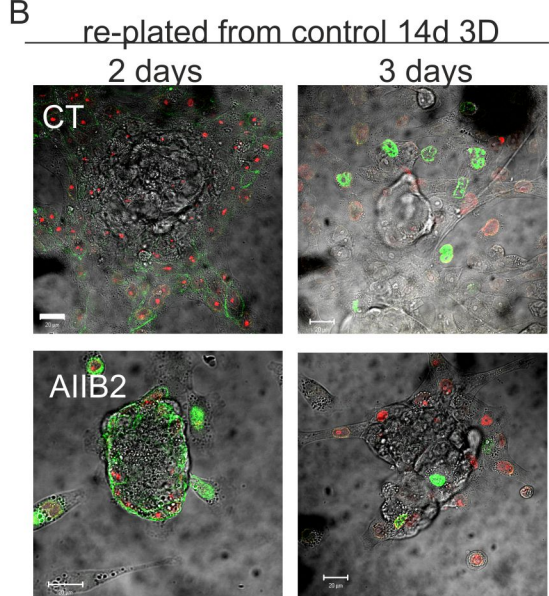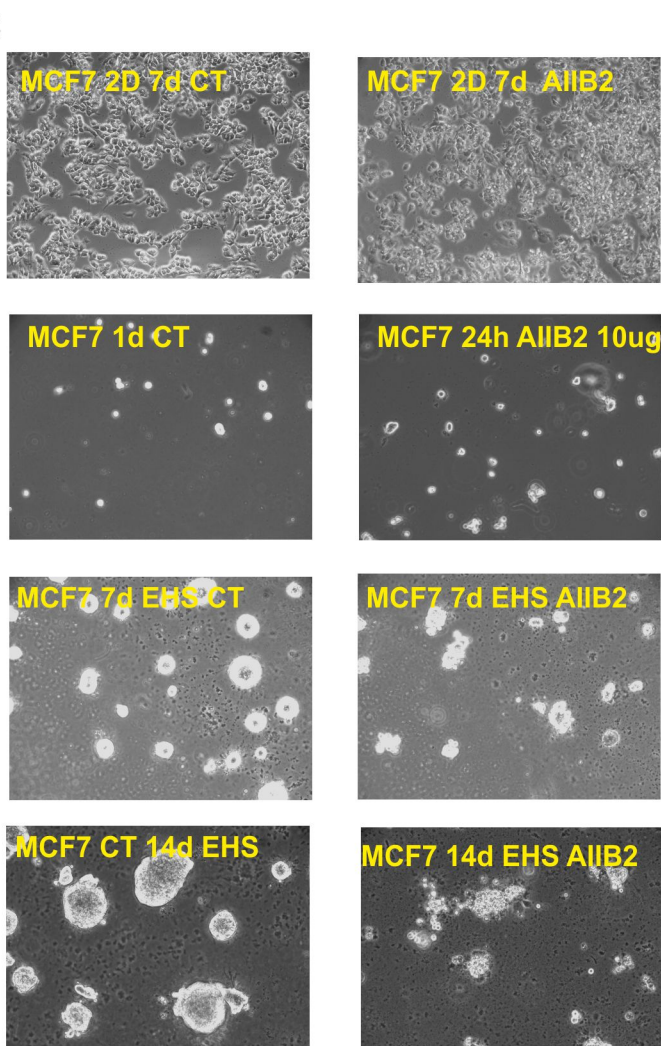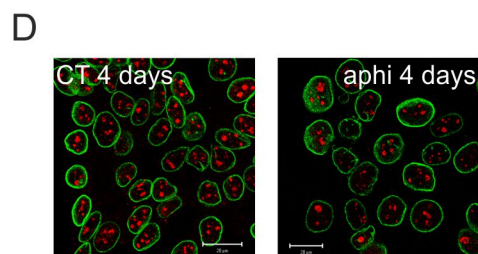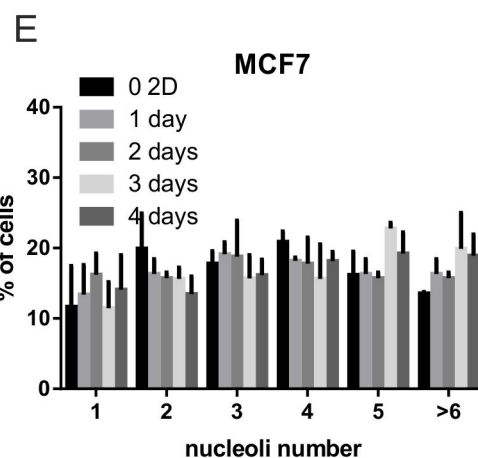

Supplement: 2015CC6907-f02-z-4c.pdf [file kccy-15-03-1121354-s001.pdf]
